# Antidiabetic Medicinal Plants Used by the Basotho Tribe of Eastern Free State: A Review

**DOI:** 10.1155/2016/4602820

**Published:** 2016-06-29

**Authors:** Fatai Oladunni Balogun, Natu Thomas Tshabalala, Anofi Omotayo Tom Ashafa

**Affiliations:** Phytomedicine and Phytopharmacology Research Group, Department of Plant Sciences, University of the Free State, Qwaqwa Campus, Private Bag X 13, Phuthaditjhaba 9866, South Africa

## Abstract

Diabetes mellitus (DM) belongs to the group of five leading important diseases causing death globally and remains a major health problem in Africa. A number of factors such as poverty, poor eating habit, and hormonal imbalance are responsible for the occurrence of the disease. It poses a major health challenge in Africa continent today and the prevalence continues to increase at an alarming rate. Various treatment options particularly the usage of herbs have been effective against diabetes because they have no adverse effects. Interestingly, South Africa, especially the Basotho tribe, is blessed with numerous medicinal plants whose usage in the treatment of DM has been effective since the conventional drugs are expensive and often unaffordable. The present study attempted to update the various scientific evidence on the twenty-three (23) plants originating from different parts of the world but widely used by the Sotho people in the management of DM. Asteraceae topped the list of sixteen (16) plant families and remained the most investigated according to this review. Although limited information was obtained on the antidiabetic activities of these plants, it is however anticipated that government parastatals and scientific communities will pay more attention to these plants in future research.

## 1. Introduction

Diabetes mellitus (DM) is an endocrine disorder marked by abnormalities in lipid, carbohydrates, and protein metabolism. It does not only cause hyperglycemia but result in numerous complications which are grouped as acute, subacute, or chronic; these include but are not limited to retinopathy, neuropathy, nephropathy, cardiovascular disorders, hypoglycemia, diabetic ketoacidosis, hyperosmolar nonketotic syndrome, polydipsia, frequent urination, lack of vigour, ocular impairment, weight loss, and excessive eating (polyphagia) [1, 2].

Diabetes mellitus (DM) may be classified based on the etiology and clinical symptoms as type 1 (insulin dependent diabetes mellitus, IDDM) and type 2 (non-insulin dependent diabetes mellitus, NIDDM). It is a typical and very predominant disease which troubles people of different classes and races worldwide [3]. Report from International Diabetes Federation (IDF) revealed that the menace presently affects well over 366 million (M) people globally and that, by 2030, the figure will be reaching 552 M [4]. It is estimated that Nigeria (3.2 M), South Africa (2 M), Kenya (over 0.7 M), and Cameroun (over 0.5 M) top the list of countries with the prevalence of the disease in each subregion of Africa [5]. DM is also considered a vital cause of disability and hospitalization as it results in significant financial burden [6].

Due to the inherent side effects such as hypoglycemia, weight increase, gastrointestinal (GIT) disturbances, nausea, and diarrhoea [7] of common oral hypoglycemic synthetic drugs like sulphonylureas (glibenclamide, e.g., Daonil), biguanides (metformin, e.g., Glucophage), and glucosidase inhibitors like Acarbose, researchers are now intensifying efforts in alternative and complementary medicines to proffer lasting solution or at least stem the burden of this menace [8]. This is partly because herbal remedies are more efficient and have little or no adverse effects and could also be due to the fact that they form a vital component of the health care delivery system in most African nations [9]. World Health Organization (WHO) in one of their submissions advocated the evaluation of medicinal plants (MP) based on their efficacy, low cost, and possession of little or no adverse effects [10]. Similarly, WHO in one of their technical reports [11] maintained that 4/5th of the citizens in African countries rely on folk medicines particularly herbal remedy for their primary health care requirements [12, 13]. This could be ascribed to the efficacy and availabilities of these plants because they account for 25% of higher plants in the world comprising 5 400 with more than 16 300 medicinal uses [14]. South Africa (SA) accounts for 9% (about 30 000 species) of higher plants in the world [15]. It is therefore not amazing that over 3 500 species of these plants are employed by over 20 000 indigenous healers [16]. Interestingly, about 80% of South Africans use plants for therapeutic purposes [17] mainly because the cost of buying orthodox medicine or conventional treatment continues to increase, thus making affordability impossible.

Herbal drugs with antidiabetic activity are known for their therapeutic potentials within the traditional healthcare system, but despite their pronounced folkloric activity, they have not been commercially formulated as modern medicines. This is despite the fact that their therapeutic properties have been reported to serve as a potential source of hypoglycemic drugs and many of these compounds derived from plants are used in the management of DM. This is confirmed by numerous ethnobotanical surveys conducted on medicinal herbs employed in the control of DM from divergent regions, communities, and tribes within the African subregion [18–27].

**Figure 1:**
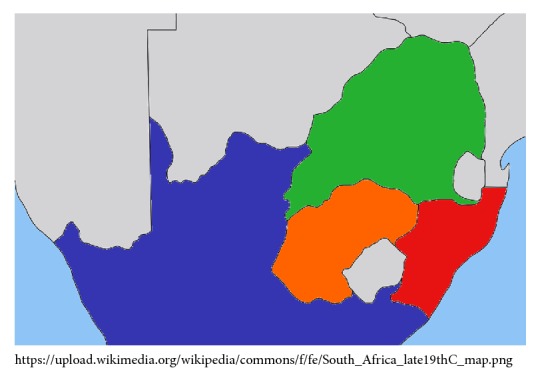
Map of South Africa showing the Basotho region (highlighted in orange).

Basotho (South Sotho) tribes are the largest population of blacks within SA and they are concentrated in Free State, Gauteng, and Eastern Cape (Figure 1) Provinces. It is worth mentioning that their knowledge and usage of numerous MP in the treatment of various disorders such as DM and hypertension cannot be overemphasized. Tshabalala and Ashafa [28] in the past conducted an ethnobotanical overview of plants utilized for diabetes control by the Basotho people and identified twenty-three (23) plants with such potentials. In this paper, we conducted a comprehensive review of these plants with a view to helping researchers and government agencies to avert the probable extinction of these plants. This review is also intended to serve as a guide for possible future research on the scientifically unproven plants.

## 2. Methodology

Literature used for this review was obtained through searching the individual botanical names of the plants on Google Scholar. Informative articles used in this study were sourced from scientific databases such as Science Direct, PubMed, and Medicine. The articles mostly cover the period between 2000 and 2015. One hundred and sixteen (116) journals were retrieved, although emphasis was placed on the hypoglycemic and/or hyperglycemic and antihyperlipidemic activities of the plants when keywords such as MP and hypoglycemic were typed in. Various other* in vitro* and* in vivo* pharmacological activities of these plants (Tables 1 and 2) were sourced from 79 of the peer-reviewed articles.

## 3. Basotho Ethnobotanically Reported Plants with Antidiabetic Potentials

### 3.1. *Eriocephalus punctulatus*

*Eriocephalus punctulatus* is a flowering plant belonging to the Asteraceae (daisy) genus family with over 35 species. It is commonly called wilde roosmaryn, Kapokbos (meaning snowbush in Afrikaans), and wild rosemary (Eng.) and it is widely distributed in SA (in mountain areas of Free State and Western Cape Provinces) and Namibia [29, 30]. Traditionally, the plant is used as diaphoretic and diuretic agents [31] and for the treatment of cold [32], DM, and so forth.* E. punctulatus* contains essential oil called Cape chamomile which comprises over 50 aliphatic esters with 2-methyl butyl-2-methyl propanoate (21.2%), 7-methylbutyl-2-methyl butanoate (5.6%), 2-methylpropyl-2-methyl propanoate (5.3%), 7-methyl-2-octyl acetate (4.5%), linalyl acetate (4.4%), and *α*-pinene (1.9%) as main compounds [29] reported to be of use in cosmetic toiletries and aromatherapy [29]. The anti-inflammatory, antiallergic, antidepressant, and antiseptic properties of* E. tenuifolius* essential oil, a related species of* E. punctulatus*, have been reported [33]. Njenga et al. [34] reported the antimicrobial activity of* E. punctulatus* with other species of* Eriocephalus* and 113 essential oils. Antioxidant and anti-inflammatory properties were also reported [35] however; a report from chemotaxonomic evidence suggests that Cape chamomile oil is a product of* E. tenuifolius* and not* E. punctulatus* [33] and to date no scientific evidence of its antidiabetic potentials has been reported.

### 3.2. *Hypoxis hemerocallidea*

*Hypoxis hemerocallidea* formerly referred to as* H. rooperi* (African potato) according to Laporta et al. [36] belongs to the Hypoxidaceae (star lily) family. The locally called star flower and yellow star (Eng.); sterblom and gifbol (Afr.); moli kharatsa and Lotsane (South Sotho); or Inkomfe (Zulu) is widely distributed within SA virtually in all the provinces and can be found in other African countries such as Botswana, Lesotho, and Swaziland. There are over 76 species of the genus* Hypoxis* in Africa, 40 of which are found in SA while 16 others are endemic to SA. Traditionally, various parts of the plant are used in the treatment of various diseases such as dizziness, burns, wounds, anxiety, depression or insanity, DM, cancer, polyarthritis, hypertension, and asthma [37, 38]. The formulated and marketed products of the species have been reported to ameliorate benign prostrate hypertrophy (BPH), urinary infections, and immune modulations [39]. Activities of this plant are attributed to its main bioactive compounds, hypoxoside and its aglycone derivative, rooperol [40]. Katerere and Eloff [41] maintained that the leaves and corms of the plant possess antibacterial and antioxidant activities while the anticonvulsant activity was recently reported by Liu et al. [42]. The cardiovascular activity of* H. hemerocallidea* was reported in experimental animal models [38], antidiarrhoea activity was reported in rodents [43], and the uterolytic effect was found in rats and guinea pigs [44]. The antinociceptive activity (studied in mice) and the anti-inflammatory and antidiabetic activities (rats) have been reported when aqueous extract (50–800 mg/kg) of this plant was administered to rodents induced with a rat hind paw oedema (0.5 mg/kg) and streptozotocin (90 mg/kg), respectively (Table 2). The herb was able to bring about a significant reduction in the fresh egg albumin-induced acute inflammation of the rat hind paw and blood glucose concentrations in these animals [37]. Several other reports of these activities had also been detailed by several authors [40, 45–47].

### 3.3. *Dicoma anomala*

*Dicoma anomala* belongs to the Asteraceae family. The plant commonly called fever bush and stomach bush (Eng.); maagbitterwortel, kalwerbossie, koorsbossie, gryshout, and maagbossie (Afr.); Hloenya and Mohasetse (South Sotho); Inyongana (Swazi, Xhosa); or Isihlabamakhondlwane and Umuna (Zulu) is a herbaceous plant that grows in grassland, stony places, hillsides, flat grasslands, and savannah areas within an altitude that ranges from 165 to 2075 m [48].* D. anomala* is widely distributed within SA in places such as Limpopo, North West, Gauteng, Mpumalanga, Free State, Northern Cape, and KwaZulu-Natal [49] and over 16 species of the genus* Dicoma* exist in SA with* D. tomentosa* and* D. anomala* being the most distributed species in Africa. Traditionally, the plant is used in the treatment of coughs and colds, fevers, ulcers, dermatosis, venereal diseases, labour pains, dysentery, intestinal parasites, stomach pains, toothache, and internal worms which are linked to versed majority of pharmacological activities such as antihelminthic, antispasmodic, analgesic, wound healing, anti-inflammatory, and antimicrobial activities [50–52].* Dicoma tomentosa* from the same genus was reported to possess antiplasmodial activity [53] while the* in vitro* inhibitory potentials of* D. anomala* against cytochrome p450 enzymes and p-glycoprotein were also reported [54]. The antiplasmodial activity of* D. anomala in vitro* [55] as well as the* in vitro* antioxidant activity is recently reported (Table 1) by Balogun and Ashafa [56] but unfortunately, report on the* in vivo* antidiabetic activity of the plant is still awaited in the scientific world.

### 3.4. *Xysmalobium undulatum*

*Xysmalobium undulatum* is a member of the Apocynaceae family. The plant is commonly called milk bush, milkwort, Uzura, wild cotton, and wave-leaved* Xysmalobium* (Eng.); bitterhout, bitterwortel, bitterhoutwortel, and melkbos (Afr.); Leshokoa and Poho-tsehla (South Sotho); iyeza elimhlophe, Nwachaba, and Ishongwane (Xhosa); or Ishongwane, Ishongwe, and Ishinga (Zulu). The plant is widely distributed in all provinces within SA as well as Namibia, Botswana, Lesotho, and Swaziland. There are over forty (40) species of the genus* Xysmalobium* in the world and 24 species of these are found in SA. Locally, the usage of the plant is in the treatment of stomach cramps, diarrhoea, colic, afterbirth cramps, headache, wounds, and abscesses [48, 50, 57, 58]. The main compounds as elucidated by Ghorbani et al. [59] are uzarin and xysmalorin with few quantities of allouzarin and alloxysmalorin. Steenkamp et al. [60] reported the* in vitro* antioxidant activity and selective serotonin reuptake inhibitors (SSRI) antidepression activity of the plant reported by Pedersen et al. [61]. Antibacterial and antifungal properties [62], antiplasmodial and central nervous system (CNS) activity [63], antidiarrhoeal activity [64], and inhibition of uptake of serotonin [16] are reported. Still, no scientific work on the antidiabetic activity of the plant was found in the literature.

### 3.5. *Morella serrata*

*Morella serrata* belongs to the Myricaceae family and it is locally called Smalblaarwasbessie and Berg-wasbessie (Afr.); lance-leaved strawberry, waxberry, and mountain waxberry (Eng.); Isibhara, Umakhuthula, and Umaluleka (Xhosa); or Iyethi, Ulethi, Umakhuthula, and Umlulama (Zulu).* M. serrata* grows along streams on grassy hillsides as well as on forest fringes. They are widely distributed within SA virtually in all the provinces while they are also occurring in Swaziland, Zimbabwe, and northern Botswana. Traditionally, the plant is used to cure headaches and tuberculosis [65] and for the management of DM. The antimicrobial and antitumor activity of the plant have been reported [66]; however, it is worth reporting that the pharmacological evidence of its antidiabetic efficacy still remains unknown.

### 3.6. *Gazania krebsiana*

*Gazania krebsiana* belongs to the family Asteraceae and it is locally referred to as terracotta* Gazania* meaning “beautiful flower” (Eng.); gousblom and botterbloom (meaning) butterflower (Afr.). There are over nineteen (19) species of the genus* Gazania* in Africa and most of these are predominantly found in SA. The plant is distributed in all the provinces of SA from Namaqualand in the west to the Eastern Cape and KwaZulu-Natal (KZN) in the east, through Free State in the north and Gauteng. The plant is used in the management of DM traditionally among the Basotho tribe and recently Balogun and Ashafa [56] reported the antioxidant activity of the plant in* in vitro* study (Table 1) but in our view, there is no scientific evidence to support its antidiabetic efficacy to date.

### 3.7. *Elephantorrhiza elephantina*

*Elephantorrhiza elephantina* is a member of Fabaceae or Leguminosae family. The plant is commonly called eland's bean, eland's wattle, and elephant's root (Eng.); baswortel, elands-boontjie, leerbossie, looiersboontjie, and olifantswortel (Afr.); Mupangara (Shona); Mositsane (South Sotho, Tswana); or Intolwane (Xhosa, Zulu). There are over nine (9) species of the genus* Elephantorrhiza* and the species* elephantina* is the most widely spread. It can be found in southern Angola, Namibia, Botswana, Zimbabwe, and Mozambique and in most provinces within SA. Traditionally, the plant is used to treat diarrhoea, dysentery, stomach disorders, haemorrhoids, peptic ulcer, skin diseases, and acne [58, 67–70]. The anthelmintic activity* in vitro* and* in vivo* was reported by Maphosa et al. [71]. Equally, the antiprotozoal activity has been reported [72, 73]. Mathabe et al. [74] also reported the antibacterial activity while the anti-inflammatory, antinociceptive* in vivo* [75], antimicrobial [76], antioxidant, cytotoxic [77], and immune enhancing as well as anti-HIV [78] activities were similarly reported (Table 1). Despite these various pharmacological reports, there has not been any scientific literature that supports its antidiabetic properties.

### 3.8. *Hermannia pinnata*

*Hermannia pinnata* belongs to the family of Malvaceae (Mallow) and is commonly called orange* Hermannia* or doll's rose (Eng.); Kwasblaar and Kruip Poprosie (Afr.). There are over 180 species of the genus* Hermannia* in SA; 162 species of these are widely spread across the country. Traditionally, the Sotho tribe used the plant in the management of DM but the scientific efficacy has not been ascertained.

### 3.9. *Commelina africana*

*Commelina africana*, a member of the Commelinaceae family, is commonly called yellow* Commelina* (Eng.); geeleendagsblom (Afr.). Sixteen (16) of the over 170 species of the genus* Commelina* in the world are found in SA. They are mostly distributed in Africa (e.g., Madagascar) as well as other places of the world such as Arabian Peninsula where there are forest, savannah, and grassland. Traditionally, the plant is useful in the treatment of venereal diseases and as medicine for women suffering from menstrual pain and infertility [31, 79, 80]. The hypoglycemic effect of the plant extract had been reported in alloxan (125 mg/kg) induced diabetic rats when aqueous leaves extracts of the plant brought down the increased glucose level of the animals (Table 2). This reduction by the plant not only was attributed to its inhibitory effect on glucose absorption but could probably be due to other mechanisms such as direct stimulation of glycolysis in peripheral tissues, facilitation of glucose entry into peripheral cells, reduced hepatic gluconeogenesis, and reduction of plasma glucagon levels [81].

### 3.10. *Haplocarpha scaposa*

*Haplocarpha scaposa* belongs to the family Asteraceae and is commonly called false gerbera (Eng.); melktou (Afr.); Khutsana (South Sotho); or Isikhali (Xhosa). Ten (10) species of the genus* Haplocarpha* existed and five (5) of them occur in central Africa.* H. scaposa* is endemic to Africa and is widely distributed in Mpumalanga, Free State, Eastern Cape within SA, Swaziland, and some part of eastern Africa [82]. Traditionally, the plant is used to reduce menstrual pain and in the management of DM [82].* H. scaposa* has been reported to exhibit anti-inflammatory activity [83]; however, it is noteworthy to report that research on the antidiabetic activity of the plant has not been validated to date.

### 3.11. *Helichrysum aureum*

The plant belongs to the Asteraceae family and is locally called Leabane (South Sotho). According to Flora of Zimbabwe [84], there are over 600 species of the genus* Helichrysum* in the world; about 244 to 250 of these species are found in Africa, particularly SA. The plant is found in submontane grassland and miombo woodland areas with a wide distribution in Mozambique, Zimbabwe, Lesotho, Swaziland, and SA (precisely Eastern Cape and Free State). The areas of the world where* Helichrysum* species predominates include southern Europe, southwest Asia, southern India, Sri Lanka, and Australia [85]. The antimicrobial and cytotoxic activity of the plant had been reported [86].

### 3.12. *Empodium plicatum*

*Empodium plicatum* belongs to the Hypoxidaceae family and is locally called golden star (Eng.); Ploegtydblommetjie (Afr.).* E. plicatum* is endemic to SA and widely distributed in Northern and Western Cape. Traditionally, Basotho people use the plant to manage DM although information from the literature at the time of compiling this review reveals no scientific report on the pharmacological activity of the plant.

### 3.13. *Mimulus gracilis*

The plant belongs to the family of Scrophulariaceae and is locally called Sehlapetsu (South Sotho). However, the plant is not endemic to SA but is widely distributed in Eastern Cape, Free State, KZN, Limpopo, Mpumalanga, Northern Cape, and North West Provinces. It is also reported to be abundant in Angola, Botswana, Namibia, Nigeria, Sudan, Kenya, Tanzania, Ethiopia, Malawi, Mozambique, Zambia, Zimbabwe, Lesotho, Yemen, India, China, and Australia. Traditionally, the plant is used to treat DM by Basotho people; there is urgent need to determine the antidiabetic activity of the plant for the treatment of DM.

### 3.14. *Pentanisia prunelloides*

*Pentanisia prunelloides* belongs to the Rubiaceae family. The plant is locally called wild verbena and broad-leaved* Pentanisia* (Eng.); Sooibrandbossie (Afr.); or Icimamlilo (Zulu). Three (3) of the 15 species of the genus* Pentanisia* are found in SA. Traditionally, various uses of the plant include diarrhoea, dysentery, rheumatism, heartburn, vomiting, fever, toothache, tuberculosis, snakebite, haemorrhoids, burns, and swellings. The* in vitro* anti-inflammatory and antioxidant activity of the plant have been reported [87]. Mpofu et al. [77] established the antibacterial, cytotoxic, and antioxidant activities while the anti-inflammatory, antimycobacterial, antimicrobial, nongenotoxic [88–90], and antioxidant and anti-inflammatory [91] activities* in vitro* had also been reported (Table 1). It is interesting to note that, despite the various* in vitro* activities, there is a dearth of information on the antidiabetic activities of the plant.

### 3.15. *Cannabis sativa*

*Cannabis sativa* belongs to the Cannabaceae family. The local names include marijuana (Eng.); dagga (Afr.); Umya (Xhosa); Matekwane or Patse (Northern Sotho); or Nsangu (Zulu). The plant originated from Asia but is presently being cultivated in many countries of the world though naturalized in SA. Three varieties of cannabis are recognized, namely,* sativa* which is commonly referred to as hemp, cultivated for psychoactive cannabinoids, durable fibre, and nutritious seed [92], while the other varieties are* indica* and* spontanea*.* Cannabis* is widely distributed in SA and* sativa* variety predominates in Botswana, Limpopo, North West, Gauteng, Mpumalanga, KZN, Western Cape, Eastern Cape, Lesotho while* indica* variety can also be found in Mpumalanga, whereas* spontanea* variety is in Northern Cape. Traditionally, the plant is used as a cure for asthma, bronchitis, headache, flu, epilepsy, cough, and pains. The crude drug and the pure chemical derivatives are used in modern day medicine in the treatment of a migraine, epilepsy, malaria, glaucoma, nausea, acquired immune deficiency syndrome (AIDS), appetite induction for cancer patients, and muscular spasm suppression in multiple sclerosis [93]. Its main active compound is called Δ^9^-tetrahydrocannabinol [94, 95]. The antipsychotic activity of the plant has been investigated and reported in rodents and humans [95–97]. The neuroprotective, antioxidative, and antiapoptotic activity [98] and the antibacterial activity of cannabinoids [99] and anticonvulsive, anti-inflammatory, and analgesic activity of Δ^9^-tetrahydrocannabinol have been reported [100–102], but till date, the scientific validation of the antidiabetic activity has not been reported.

### 3.16. *Bulbine narcissifolia*

*Bulbine narcissifolia* is a member of the Asphodelaceae family. The local names include strap-leafed bulbine and snake flower (Eng.); lintblaar bulbine, geelslangkop, and wildekopieva (Afr.); Khomo-ea-balisa and Serelelile (South Sotho). It has been reported that different names of the plant are adopted based on the appearance and due to the wide use of the genus by all stakeholders or tribes within SA [14, 93]. There are 73 species of the genus* Bulbine*; 67 are predominant in Africa while 6 species are found in Australia. The most common species are* B. frutescens*,* B. abyssinica*,* B. latifolia*,* B. natalensis*, and* B. narcissifolia*. The latter species is widely distributed in Western Cape, Eastern Cape, Free State, KZN, North West, Gauteng, and Limpopo within SA and in Lesotho, Botswana, and Ethiopia. Traditionally, the plant is of utmost importance in wound healing and as a mild purgative [103] and in vomiting, diarrhoea, urinary infections, DM, rheumatism, and blood-related problems. The antibacterial [104] and anticancer and antimicrobial [105] activities of the plant have been reported* in vitro* but till date, there has not been any scientific evidence of antidiabetic properties.

### 3.17. *Rumex lanceolatus*

*Rumex lanceolatus* belongs to the Polygonaceae family; the common names include the small dock, smooth dock, and common dock (Eng.); Gladdetongblaar (Afr.); Idolo Lenkonyane (Zulu); Idolonyana (Xhosa); Khamane, Kxamane, and Molokoli (South Sotho). The plant is not endemic to SA but is widely distributed within SA in Eastern, Western, and Northern Cape, Free Sate, Gauteng, KZN, Limpopo, Mpumalanga, and North West. Ethnobotanically, the root and rather the leaves are used as medicine [106]. The plant serves as a cure for infertility, intestinal parasites [31], internal bleeding [107], and DM. The nonmutagenic activity [108] has been reported while the presence of chrysophanol and related glycosides has been attributed to its laxative activity [107]; however, there has not been any scientific fact about its antidiabetic efficacy in the literature.

### 3.18. *Gunnera perpensa*

*Gunnera perpensa* is one of the members of Gunneraceae family. It is locally referred to as river pumpkin and wild rhubarb (Eng.); rivierpampoen and wilde ramenas (Afr.); Qobo (Sotho); Uqobho (Swati); rambola-vhadzimu and shambola-vhadzimu (Venda); Iphuzi lomlambo and Ighobo (Xhosa); Ugobhe and Ugobho (Zulu). Fifty (50) species of the genus* Gunnera* existed and only* perpensa* are found in Africa.* Gunnera* are naturally occurring in central and southern Africa, Madagascar, New Zealand, Tasmania, Indonesia, Philippines, Hawaii, Mexico, and central and southern America.* G. perpensa* is widespread in Sudan, Ethiopia, Zaire, Rwanda, Uganda, Kenya, Zimbabwe, SA (Western and Northern Cape), Swaziland, Lesotho, Namibia, and Botswana [109]. Traditionally, the plant is used to remove placenta in newborn and to relieve menstrual pain [106, 110–112]. The toxic effect of the plant was investigated in rats when aqueous extract of the plant at different concentrations (50–400 mg/kg body weight) was administered and 20% mortality was observed in subacute (400 mg/kg dose) and chronic toxicity (200 mg/kg) tests indicating the toxic effect of the plant over long usage [113]. The antifungal and antibacterial [60, 62, 114], antioxidant [60, 115], antimicrobial [116], anthelmintic [117], and uterotonic activity [118]* in vitro* have been reported (Table 1). Moreover, the lactogenic activity* in vivo* was investigated and reported in female rats over 400–1600 mg/kg concentration ranges [119] but despite the various pharmacological effects of the plant* in vitro*, the antidiabetic activity had been reported.

### 3.19. *Aloe vera*

The plant belongs to the Liliaceae family and has its origins in Africa. The plant is commonly called Indian Aloe, True Aloe, Barbados Aloe, Burn Aloe, and so forth [120].* Aloe vera* is widely distributed in places such as Arabian Peninsula, Morocco, Mauritania, and Egypt. Traditionally, the plant is used in the treatment of various ailments which includes stimulation of cell growth, restoration of damaged cells, restoration of damaged stomach mucous membrane [121], alleviation of various gastrointestinal tract (GIT) disturbances, haemorrhoid treatment [122], wound healing, thermal burn or sunburn [123], and body immune system stimulation [124]. The anti-inflammatory [125–128], modulatory, antiprotozoal, ultraviolet (UV) protective [128], antimicrobial [121], and antifungal [129] activity* in vitro* have been reported (Table 1). The wound healing, hypoglycemic, hypolipidemic, and antioxidant activities of the plant* in vivo* in rabbit and rodents [130–132] have been reported (Table 2).

### 3.20. *Asparagus asparagoides*

*Asparagus asparagoides* is a member of Asparagaceae family. The plant is locally called African* Asparagus* fern, baby smilax, bridal creeper, and so forth.* A. asparagoides* is native to SA, Lesotho, and Swaziland and widely distributed in southern Australia and Europe, New Zealand, Hawaii, and California. Traditionally, it is used by Basotho tribe of eastern Free State in the management of DM, though; there has not been any scientific proof for this folkloric use till date.

### 3.21. *Anthospermum ternatum*

The plant is a member of Rubiaceae family. It is widely distributed in Angola, Malawi, Zambia, Zimbabwe, and Tanzania. No scientific report of its antidiabetic activity has been reported to date despite its usage by the Basotho people in the management of DM.

### 3.22. *Erythrina zeyheri*

*Erythrina zeyheri* also belongs to Fabaceae family and Faboideae subfamily. The plant is commonly called harrow-breaker and plough-breaker (Eng.); Ploegbreker (Afr.); Khungoana and Motumo (South Sotho); Umnsinsana (Zulu). The plant grows in grassland and moist vleis with clay soils or sandy soils and it is found in colonies. It is widely spread in Mpumalanga, North West, Gauteng, Free State, KZN, and Lesotho. Traditionally, the plant has its usage in asthma, tuberculosis, rheumatism [133], and DM treatments. The antibacterial [134, 135] and anti-inflammatory [133] activity of the plant have been reported, though; scientific evidence on the antidiabetic activity is still awaited.

### 3.23. *Sisymbrium thellungi*

*Sisymbrium thellungi* belongs to the* Brassicaceae* (Cabbage) family and is commonly called African turnip weep. The plant is native to SA and widely distributed in Northern New South Wales, Queensland, and eastern part of SA. The scientific evidence on antidiabetic potentials of the plant has not been submitted to date.

## 4. Conclusion

Diabetes mellitus (DM) is a major endocrine disorder and its growth or prevalence is attributed to a number of factors that include but are not limited to obesity, social structure, hormonal imbalance, and hereditary. The current trend in the management of DM characterized by hyperglycemia involves the use of herbs since the oral hypoglycemic agents (OHAs) are known to result in unwanted side effects; hence, the need to explore rich and potential plants with antidiabetic activity became necessary. However, from our review, it is evident that the folkloric use of most of these MP has not been adequately explored, thus the need for the government to sponsor or support more research activities in this area so that the potential in these plants to offer lasting solutions to the management of this menace can be ascertained.

## Figures and Tables

**Table 1:** * In vitro* pharmacological activities of medicinal plants used in the treatment of diabetes mellitus by the Basotho tribe.

Plant name	Family	Local name (South Sotho)	Pharmacological studies				Solvent used	Province	Part used	References
			*Antioxidant*	*Inflammation*	*Antimicrobial*	*Cytotoxic*				
*E. punctulatus*	Asteraceae	Kapokbos (Afr.)	DPPH	5-Lipoxygenase enzyme	Disc diffusion assay	*∗*	Acetone		Leaves	[34, 35]

*H. hemerocallidea*	Hypoxidaceae	Lotsane	DPPH	*∗*	Microdilution assay	*∗*	Ethanol, acetone	Mpumalanga	Leaves and corms	[41]

*D. anomala*	Asteraceae	Hloenya	DPPH, ABTS, hydroxyl radicals, superoxide anion, metal chelating, reducing power	*∗*	*∗*	*∗*	Water, ethanol, methanol, and 50% aqueous ethanol	Free State	Roots	[56]

*X. undulatum*	Apocynaceae	Leshokoa	*∗*	*∗*	Microplate dilution method	*∗*	Water, ethanol, ethyl acetate	KZN	Roots	[62]

*M. serrata*	Myricaceae	Smalblaarwasbessie (Afr.)	*∗*	*∗*	Microplate dilution method	Brine shrimp lethality assay	Hexane, water, methanol, acetone	Free State	Roots	[66]

*G. krebsiana*	Asteraceae	Botterbloom (Afr.)	DPPH, ABTS, hydroxyl radicals, superoxide anion, metal chelating, reducing power	*∗*	*∗*	*∗*	Water, ethanol, methanol, and 50% aqueous ethanol	Free State	Leaves	[56]

*E. elephantina*	Fabaceae	Mositsane	DPPH	*∗*		Brine shrimp lethality assay	Hexane, water, ethanol	Gauteng	Rhizomes	[77]
					Microdilution method		Water, DCM/water	Swaziland, South Africa, and Zimbabwe	Rhizomes	[76]

*H. scaposa*	Asteraceae	Khutsana	*∗*	Cyclooxygenase	Disc diffusion	*∗*	Hexane, methanol, water	Pietermaritzburg	Leaves, roots	[83]

*P. prunelloides*	Rubiaceae	Sooibrandbossie (Afr.)	DPPH	15-LOX	Microdilution assay	Brine shrimp lethality assay	Hexane, water, ethanol water, 80% ethanol, DCM	Gauteng KZN	Rhizome Whole plant	[77] [89, 91]

*B. narcissifolia*	Asphodelaceae	Serelelile	*∗*	*∗*	Cup plate method	*∗*	Water, acetone, ethyl acetate	KZN	Leave, roots, rhizome	[104]

*G. perpensa*	Gunneraceae	Qobo			Microplate dilution method		Water, ethanol, ethyl acetate	KZN	Roots	[62, 114, 116]
			DPPH, ABTS, nitric oxide, hydroxyl radicals, superoxide anion	LOX activity		Brine shrimp lethality assay	Methanol	KZN	Rhizome	[115]

*Aloe vera*	Liliaceae	Barbados Aloe	Peroxyl radical, superoxide		Agar diffusion Agar dilution		Water	Ondo, Nigeria Romania	Leaf, gel Leaves	[121] [128, 129]

*E. zeyheri*	Fabaceae	Khungoana			Agar dilution		Acetone	Japan	Roots	[134, 135]

^*∗*^The pharmacological activity yet to be determined.

DPPH: 1,1-diphenyl-2-picrylhydrazyl; ABTS: 2,2-azino-bis(3-ethylbenzothiazoline)-6-sulfonic acid.

KZN: KwaZulu-Natal; DCM: dichloromethane; LOX: lipoxygenase.

**Table 2:** List of scientifically investigated medicinal plants with *in vivo* antidiabetic activity used in Basotho traditional medicine.

Plant name	Family	Local name	Type of effect	Model	Medium/part	Dosage (mg/kg)	Province/area	References
*H. hemerocallidea*	Hypoxidaceae	Lotsane	Cardiodepressant, hypotensive, anti-inflammatory, antioxidant, hypoglycemic	Rat	Aq. stem bark, Aq.	50, 100, 200, 400	KZN	[37]
					Corms	25, 50, 100, 200, 400	*∗*	[38]

*E. elephantina*	Fabaceae	Mositsane	Analgesic, anti-inflammatory	Rat	Root	50, 100, 200	Eastern Cape	[75]

*C. africana*	Commelinaceae	Geeleendagsblom	Antihyperglycemia	Rat	Leaves	500	Ibadan, Nigeria	[81]

*Aloe vera*	Liliaceae	Barbados Aloe	Wound healing, hypoglycemic	Rabbits	Gel	*∗*	*∗*	[130]
			Hypolipidemic	Mouse	Gel	25, 50, 100	S. Korea	[131]
			Antioxidant, hypolipidemic	Rats	Gel	300	India	[132, 136]
			Hypolipidemic	Mice	Gel	350	*∗*	[137]
			Hypoglycemic, hypolipidemic	Humans	Powder	100, 2000	Ludhiana	[138]

^*∗*^Not indicated.

KZN; KwaZulu-Natal.
